# The astrolabe of molecular allergology

**DOI:** 10.1186/2045-7022-4-S2-P62

**Published:** 2014-03-17

**Authors:** Djoumouai Belaid

**Affiliations:** 1 private allergist, 19 Cité du 20 aout 1955 La Verdure Batna, Batna, 05000, Algeria

## 

To conceive and realize a practical device to help the diagnosis of molecular allergology which I named "the astrolabe of molecular allergology" nine allergological parameters incorporate this idea. The progress realized in the domain of recombined allergens (RA) has led to the appearance of a new concept in the diagnosis of allergy, the molecular diagnosis. It permits to identify molecules potentially responsible of the blossoming of diseases, and to think of molecular families rather than biological ones, allergenic constituents now classified in families of proteins based on their structure and function: A new diagnosis step came to enrich the research in Allergology. The targeted dosage of RA appeals to the data of the molecular diagnosis that the allergist is under the obligation to master. The optimization of the sensibility, the standardisation of allergens and the examination of the possible strategies of prevention of allergic diseases becomes possible: The allergist can manipulate this new data after the dosage of specific IgE, before starting up the desensitization, the oral provocation test or proposing an eviction. The objective of this work is to furnish an adequate pedagogical support, pertinent and perfectible, which, validated, may accompany the allergist in his diagnosis quest. Thanks to the data well listed of molecular allergology, this tool will permit to better delimit the crossed reactions, the hard cases of multi sensibilizations, to take the appropriate desensitization decision and to adapt supplanting diets. The molecular diagnosis of allergy is a new vision that each allergist must know and tame perfectly. Our aim is to accompany him in this progress by proposing this humble practical device. The astrolabe of molecular allergology shows us the way.

